# Zinc in Dog Nutrition, Health and Disease: A Review

**DOI:** 10.3390/ani11040978

**Published:** 2021-04-01

**Authors:** Ana Margarida Pereira, Margarida R. G. Maia, António José Mira Fonseca, Ana Rita Jordão Cabrita

**Affiliations:** LAQV, REQUIMTE, Instituto de Ciências Biomédicas de Abel Salazar, Universidade do Porto, R. Jorge Viterbo Ferreira nº 228, 4050-313 Porto, Portugal; anamargaridabp@hotmail.com (A.M.P.); mrmaia@icbas.up.pt (M.R.G.M.); ajfonseca@icbas.up.pt (A.J.M.F.)

**Keywords:** zinc, dog, bioavailability, biomarkers, requirements, zinc status-disorders

## Abstract

**Simple Summary:**

This work compiles the current state of knowledge regarding zinc requirements of healthy dogs and biomarkers of zinc status. To ensure an adequate zinc status, it is important to know the zinc content of foods and their bioavailability to assess the need and the ideal supplementation strategy regarding levels and sources of additives in complete dog foods. As zinc is required for enzymatic, structural, and regulatory functions in the animal body, its nutritional status has been associated with several pathologies that may be due to, or exacerbated by, a deficit of dietary zinc supply.

**Abstract:**

Zinc is an essential trace element, required for enzymatic, structural, and regulatory functions. As body reserves are scarce, an adequate zinc status relies on proper dietary supply and efficient homeostasis. Several biomarkers have been proposed that enable the detection of poor zinc status, but more sensitive and specific ones are needed to detect marginal deficiencies. The zinc content of commercial dry dog foods has great variability, with a more frequent non-compliance with the maximum authorized limit than with the nutritional requirement. The bioavailability of dietary zinc also plays a crucial role in ensuring an adequate zinc status. Despite controversial results, organic zinc sources have been considered more bioavailable than inorganic sources, albeit the zinc source effect is more evident after a restriction period of dietary zinc. Many disorders have been associated with inadequate zinc status, not being clear whether the occurrence of the disease is the consequence or the cause. This review presents data on zinc requirements and biomarkers for zinc status, that can be applied for the development of supplementation strategies of zinc in complete pet foods. Moreover, it provides an understanding of the role zinc plays in the health of dogs, and how altered zinc status affects diseases in dogs.

## 1. Introduction

Zinc has an acknowledged essential biological role for all living organisms. The importance of zinc for the metabolism of microorganisms was discovered in 1866 when Jules Raulin confirmed its essentiality for the growth of *Aspergillus niger* [1]. However, its essentiality for mammals was only recognized in 1934 for rats [2], in 1961 for humans [3], and in 1962 for dogs [4]. Under physiological conditions, the dogs’ access to zinc depends upon oral intake. To ensure an optimal zinc status and simultaneously meet dog requirements and legal limits for zinc content, zinc bioavailability additives in pet foods is of utmost importance. In this sense, recent studies have evaluated inorganic and organic sources of zinc at different levels of inclusion to enhance zinc bioavailability in dog foods [5,6,7,8,9,10,11,12,13]. The optimal zinc status of dogs promotes health, wellbeing, and increases lifespan.

Indeed, an adequate zinc status is required for the proper function of several systems. In dogs, the role of zinc in skin health has been well documented [14,15,16]. Besides, clinical reports show that impairment of zinc status might be associated with disorders in other systems. However, to fully comprehend the mechanisms that link zinc to the clinical signs and laboratory findings reported in dogs, it is necessary to acknowledge zinc functions at cellular and molecular levels. As such studies are scarce in dogs, resorting to data extrapolation from other mammals is often required. Moreover, while heavily deficient zinc status often has clinical manifestations, more specific biomarkers are required to differentiate marginal deficiencies and optimal levels, which constitute an additional challenge.

The purpose of this review is to describe the importance of zinc for dogs and highlight the role of proper dietary zinc supply. It is intended to provide insights into the practical aspects of zinc nutrition, including assessment of zinc biomarkers, crucial to define nutritional requirements for healthy individuals, compliance of commercial dog foods with requirements and maximum permitted levels, as well as supplementation strategies to enhance zinc bioavailability. Moreover, the state of knowledge regarding zinc in the health and disease of dogs will be presented and confronted with studies from other mammals, to allow a better and integrative understanding of the available data.

## 2. Overview of Zinc Homeostasis and Metabolism

**Figure 1:**
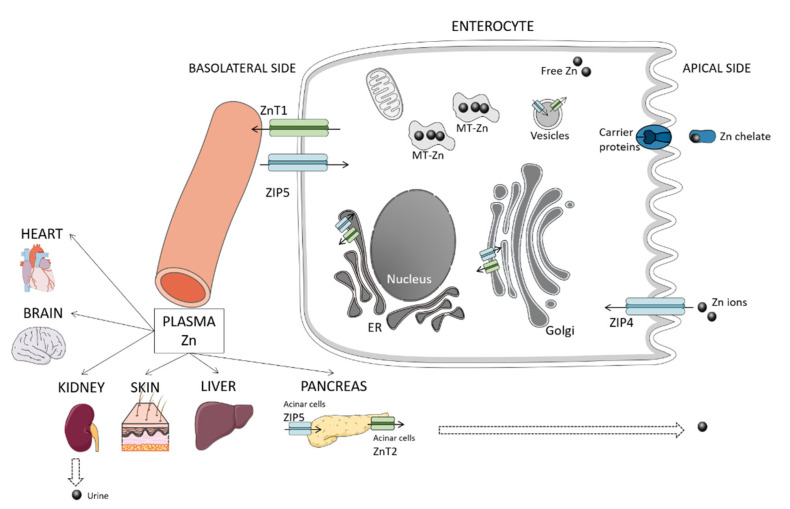
Simplified scheme of zinc metabolism: Intestinal absorption and excretion. ZIP and ZnT transporters of enterocyte membranes and intracellular components are represented in blue and green, respectively. Some of the organs that exchange zinc with plasma pool are represented. Routes of excretion are represented with dashed arrows. ER—endoplasmic reticulum; MT—metallothionein. Own source, based on References [23,24].

Zinc is predominantly distributed intracellularly (50% in the cytoplasm, 10 to 20% in membranes, and the remaining in cellular compartments that constitute zinc pools, e.g., mitochondria, endoplasmic reticulum, and Golgi apparatus) [17], serum zinc only constituting a small fraction (0.1%) of whole-body zinc [18]. Both zinc cellular influx and efflux are regulated by two zinc transporter families, the ZnT (solute carrier (SLC)30) family that lowers the cellular zinc concentration, and the ZIP (Zrt- and Irt-like protein, SLC39) family that increases cellular zinc [19]. Moreover, ZnT and ZIP are responsible for transporting zinc between cytosol and intracellular organelles/vesicles [17]. In mammals, 10 ZnT and 14 ZIP proteins have been identified and play multiple roles as their genes are expressed in several tissues and cell types [18]. The ZnT proteins are mostly found in intracellular compartments, apart from ZnT1 that is ubiquitous at plasmatic membranes, whereas the location of ZIP proteins may vary according to zinc availability and physiological conditions [20]. Additionally, metallothioneins (MT), which are low molecular weight proteins and rich in sulfhydryl groups from cysteine, have an affinity for metals and bind zinc to buffer it under physiological conditions [21]. In this way, MT participates in the regulation of intracellular storage and release, respectively, when zinc is required or depleted [21]. Both MT and other key proteins, such as ZnT1, are controlled by the metal-response element-binding transcription factor-1 (MTF-1), which acts as a cellular zinc sensor [22]. Figure 1 illustrates zinc metabolism, briefly described in the following subsections. A detailed description of zinc homeostasis in mammals can be found elsewhere [17,23,24].

### 2.1. Absorption

Dietary zinc is mostly absorbed in the small intestine, being the major absorption site in dogs, the duodenum, followed by the distal ileum and proximal jejunum [25]. The absorption mainly occurs via apical transport with the participation of zinc transporters located at the apical membrane [21]. To a small extent, zinc absorption can occur via basolateral transport, particularly in the presence of high luminal concentrations [21]. Carrier proteins may also uptake zinc as chelates [26].

In mammals, zinc transporters that mediate the transport by enterocytes include ZIP 4, ZIP 5, ZnT1, and ZnT7 [21]. ZIP4 is essential for the uptake of dietary zinc in the apical membrane. After uptake, zinc is transferred to the portal blood by ZnT1 on the basolateral membrane. Shreds of evidence show that the zinc level regulates the expression of ZIP4 in cells at a posttranslational level [27]. Although no data is available in dogs, a study performed with jejunal epithelial cells from piglets’ tissue showed increased mRNA expression of ZnT1, lower of ZIP4 and both higher mRNA expression and protein abundance of MT in groups fed a high concentration of zinc (2425 mg/kg) comparing to low (57 mg/kg) and adequate (164 mg/kg) groups, which did not differ from each other [28]. Moreover, in the study of Martin et al., the group fed low zinc had decreased expression of ZnT2 in comparison to adequate and high groups, which did not differ from each other [28]. This transporter is present in vesicles and secretory glands in the small intestine and other organs, e.g., kidney, liver, pancreatic acinar cells [18], also contributing to zinc homeostasis and responding to dietary zinc. ZnT7, which is present in enterocytes and many other tissues, e.g., liver, kidney, spleen, heart [21], transports free zinc into the trans-Golgi network, after endocytosis [21]. In mice, ZnT7 was found important for the control of zinc absorption, as animals lacking this gene had an impaired zinc status unresponsive to increases in dietary zinc [29]. Besides being transported into organelles, zinc can be bound to cytoplasmic-binding proteins, such as MT [21].

### 2.2. Excretion

Large amounts of zinc are secreted into the luminal gut through pancreatic and biliary excretions, after meals. However, most of it is reabsorbed, comprising the primary mechanism of zinc homeostasis. Acinar cells express ZIP5 in the basolateral membrane in polarized cells and can act as a passive carrier of zinc destined for secretion [19]. Moreover, ZnT5B is a bilateral transporter, located in the apical membrane of enterocytes that mediates both zinc efflux and influx [30]. Another possible route for releasing endogenous zinc into the luminal gut is the paracellular route [21].

The origin of endogenous intestinal zinc is not entirely understood, but it constitutes a source of bioavailable zinc for reuse after reabsorption or for incorporation into the resident microbiota [19]. Urinary losses of zinc occur, albeit the urinary route is not particularly important in healthy individuals. The increase of zinc in urine has been related to the catabolism of its carrier proteins [31].

## 3. Zinc Requirements and Biomarkers

### 3.1. Dietary Requirements for Healthy Individuals

Zinc was classified as probably essential for dogs in 1962—setting a requirement in dry food of 1 mg/1000 kcal of metabolizable energy (ME) [4]. In the 1974’s publication, the National Research Council (NRC) revised the zinc requirements, increasing it ten times for 12.5 mg/1000 kcal ME [32]. In the NRC’s most recent publication, the recommendation of zinc in dog foods was set at 15 mg/1000 kcal ME for adults, 25 mg/1000 kcal ME for puppies, and 17 mg/1000 kcal ME for females in gestation and peak lactation (Table 1) [33]. The European Pet Food Federation (FEDIAF) issues periodic publications with minimum recommended allowances for commercial pet food (Table 1). The values consider the NRC recommendations assuming a medium-sized lean dog with 15 kg of bodyweight, with a correction for lower energy intake, and are expressed in units per kg of dry matter (DM) or 1000 kcal of ME, based on an average daily energy intake of either 95 kcal/kg^0.75^ (398 kJ/kg^0.75^) or 110 kcal/kg^0.75^ (460 kJ/kg^0.75^). Both units are presented in scientific publications and labels of commercial products. However, for formulation purposes, it is useful the units per kg of ME, since the dietary food intake is based on the energy content of foods and metabolizable energy requirements of dogs [33]. As Table 1 displays, FEDIAF proposes minimum recommended levels higher than the adequate recommendations suggested by the NRC. It is argued that NRC recommendations have been established based on studies using purified diets with high availability and digestibility, which is often not the case of commercial ingredients in pet foods, given the nature and diversity of raw materials used [34]. Therefore, to compensate for the potential “compromised” bioavailability of nutrients in commercial foods, FEDIAF updated the recommendations, having also used scientific evidence from subsequent studies published.

### 3.2. Biomarkers of Zinc Status

The definition of zinc requirements for dogs was based on the assessment of zinc status through the evaluation of biomarkers. Zinc biomarkers enable the detection of poor zinc status and the response to dietary levels, thus constituting an important tool for planning supplementation of dog foods. Several biomarkers have been proposed with different levels of invasiveness, which is important to consider when selecting the parameters and assessment methodologies [36]. However, finding sensitive and specific biomarkers that can detect subnormal zinc status or marginal deficiencies is challenging.

#### 3.2.1. Concentration of Zinc in Serum, Tissues, and Excretions

The nutritional assessment comprehends a first screening evaluation in which it is possible to identify the risk of inadequate zinc status, e.g., enquiring owners about diet and feeding practices [37]. This provides information on daily zinc intake, which can be estimated from the dietary zinc content of food and feed intake. Low dietary zinc intake can reflect an actual quantitative flaw, in which total zinc content is insufficient to cover the needs of the dogs, favoring the onset of deficiencies. In turn, high dietary intake requires a more careful interpretation, as the consequences will critically depend on zinc bioavailability. Besides, there are no studies indicating levels of tolerance and the consequences of prolonged exposure to relatively high levels of dietary zinc, which limits drawing conclusions. The daily intake is insufficient to evaluate zinc status, as the amount indeed absorbed and used for body functions can be affected by several factors related to the food and the individual. However, the presence of clinical signs, associated with an inadequate intake of zinc, certainly raises a flag.

#### 3.2.2. Zinc Dietary Intake

Although zinc in serum represents only a small fraction of total body zinc, its concentration is often used as a marker of severe zinc deficiency, whereas for marginal or moderate deficiencies, it seems to be less sensitive [38]. Values below the normal range (0.70–2 ppm), associated with symptoms, may indicate a deficiency, whereas concentrations >5 ppm confirm toxicosis [39]. One study report increases in plasma concentration of zinc proportionally with the dietary intake [5]. However, in another study, zinc was significantly lower in plasma of dogs fed low dietary zinc (≈50 mg/kg DM), but no differences were observed between dogs fed 97 and 125 mg/kg DM (concentration of zinc analyzed) [12]. Moreover, a work of Booles et al. with postweaning puppies fed two different dietary zinc levels (50 and 200 g/kg DM), from dams that have received an adequate level of zinc during pregnancy and lactation (100 g/kg DM), did not exhibit differences in deproteinized plasma zinc concentration, questioning the sensitivity of plasma/serum zinc as a biomarker [40].

There are no reference values for zinc excretion in feces, but Ozipinar et al. observed an increase in the fecal concentration of zinc in response to dietary intake [5]. Additionally, when mature female Beagles were fed complete diets supplemented with 120 ppm of zinc from zinc methionylglycinate or zinc sulfate, fecal excretion was significantly lower in dogs supplemented with zinc methionylglycinate (11.1 mg/day) than with zinc sulfate (12.4 mg/day), reflecting the higher zinc absorption with zinc methionylglycinate [41]. As zinc is mainly excreted through feces, the determination of fecal zinc might be a useful biomarker for zinc bioavailability [42], though a tracer technique (e.g., radioactive and stable isotopes) is required for distinguishing the non-absorbed fraction from endogenous zinc losses [43].

Urine is not the preferable route for zinc excretion as this element is reabsorbed in proximal and distal renal tubules [44]. However, a study showed that urinary zinc concentration linearly increases with higher dietary intakes [5], which indicates that it may be a useful biomarker to compare levels of supplementation.

The concentration of zinc in the liver of healthy dogs averages 140 ± 25 mg/kg dry weight [45] and ranges from 10,150–54,707 mg/kg fresh weight [46], whereas in the renal cortex and renal medulla, values range from 4770–40,808 and 2805–18,828 fresh weight, respectively [46]. In other species (e.g., pigs), the concentration of zinc in these tissues responded to zinc supplementation [47]; however, no studies are available in dogs. Even though zinc concentrations in the liver and kidney may respond to zinc supplementation, its assessment requires a biopsy, which due to high invasiveness, poses problems and ethical concerns to be routinely used in dogs.

The content of zinc in hair has the advantage of being non-invasive, but its efficacy as a trustful biomarker for dogs has been controversial [16]. Booles et al. reported no response in zinc content in hair from dogs fed diets with different levels of zinc [40]. However, some authors observed that zinc content in hair reflected the bioavailability of different sources, increasing with it [7,9,40].

It is important to note that the plasma compartment exchanges with kinetically distinct zinc tissue pools, a “tissues fast”, a “tissues slow”, and a “tissues very slow”, with turnover rates within hours, days, and weeks, respectively [48]. Moreover, different intracellular mechanisms of zinc homeostasis condition the tissue accumulation of zinc [47]. Therefore, using tissue concentration for evaluation of zinc status requires taking into consideration time for distribution to evaluate, for instance, zinc repletion in deficient states or to evaluate bioavailability zinc sources in healthy individuals.

#### 3.2.3. Metallothioneins

Metallothioneins constitute intracellular zinc storage, also present in serum at low concentrations, which appear to correlate with zinc intake in some species [38]. Indeed, studies in humans showed that mRNA expression of MT in leucocytes and erythrocytes increased with zinc supplementation in 10 and 8 days, respectively, remaining elevated through the duration of treatment [49,50], whereas an induced marginal deficiency of zinc for 10 weeks induced a decrease in lymphocyte mRNA expression, with a return to baseline values after repletion [51]. Conversely, in weanling puppies, blood MT gene expression did not respond to different levels of zinc supplementation, 50, 100, and 150 mg/kg DM for 20 days, after an 8-day adaptation with a control diet (50 mg/kg DM) [12]. Similarly, plasma MT does not seem to reflect zinc intake as in a study by Booles et al., it did not respond to different levels of zinc supplementation (50 and 200 mg/kg DM) when tested in postweaning puppies, previously fed an adequate zinc level [40]. There is no data on the length of time for circulant MT or mRNA expression in blood cells to respond to variations in zinc intake of dogs; therefore, it is not possible to exclude that the lack of response could be related to the duration of the study, or if dogs are indeed more resilient towards zinc depletion than humans. Moreover, both studies were performed on puppies, which raises the question of the effect of age in MT response.

Liver MT concentration showed a positive response to dietary zinc supply in poultry and sheep [52,53] and mucosal MT in chicks [54]. In dogs, the invasive nature of the technique for tissue collection compromises the use of tissue MT concentration as a zinc biomarker.

#### 3.2.4. Zinc-Dependent Enzymes

Superoxide dismutases (SOD) are enzymes that catalyze the dismutation of superoxide radicals to molecular oxygen and hydrogen peroxide, providing cellular defense against reactive oxygen species [55]. There are four classes of SOD, iron SOD (FeSOD), manganese SOD (MnSOD), copper-zinc SOD (Cu/ZnSOD), and nickel SOD (NiSOD), being Cu/ZnSOD, the most abundant in the cytosol and extracellular space [56]. The activity of plasmatic Cu/ZnSOD was already used as a zinc biomarker, for instance, in pigs [57], showing differences between supplemented and unsupplemented diets, but not within levels of supplementation. In dogs, Cu/ZnSOD was not different when adequate levels of two zinc sources, with presumably different bioavailability, were supplemented [13], which seems to agree with the previous study in which the biomarker was not effective in marginal deficiencies and optimum zinc status.

Alkaline phosphatase, a dimeric zinc-containing phosphomonoesterase that hydrolyzes phosphate monoesters, is widely distributed in plasma and mammalian tissues, including intestines, kidneys, placenta, and bones [58]. Serum alkaline phosphatase concentration in lambs and pigs has been reported to be affected by dietary zinc levels [57,59], but no differences were found when broilers were fed diets without or with zinc supplementation [60]. In puppies, total alkaline phosphatase was higher when dogs were fed a diet supplemented with a high level (150 mg/kg) of a more bioavailable zinc source, in comparison to a low level (50 mg/kg); however, no differences were seen in the alkaline phosphatase isoenzyme profile (cortisol, liver or bone) [12]. This indicates that alkaline phosphatase might be a suitable biomarker to detect zinc deficiencies.

#### 3.2.5. Other Proteins

C-reactive protein (CRP) is an acute-phase protein secreted during an inflammation process that, in humans, has been inversely correlated to zinc concentration in plasma [61]. It showed the potential to be a biomarker, as zinc supplementation lowered serum CRP concentration of rabbits fed high-fat diets that induced disturbances in lipid metabolism [62]. Moreover, in healthy chickens fed balanced diets, serum basal CRP responded to zinc supplementation source [63]. However, in a study performed with healthy dogs fed diets supplemented with two zinc sources at similar adequate levels, CRP did not show differences, which does not explain if the bioavailability of the sources was indeed different, or if it is not a sensitive biomarker to be used in dogs [13].

## 4. Zinc in Dog Foods

### 4.1. Legal Limits and Authorized Sources of Zinc for Animal Feed Supplementation

Supplemental sources of zinc (additives) for use in animal feeds, including companion animals, require authorization in the European Union (EU). Moreover, when added, pet food producers have to comply with the legal limits concerning the total content of zinc in feeds described in Appendix 4(II) of Regulation 1831/2003/EU [64]. In addition to compliance with legal limits, pet food manufacturers are obliged to declare additives, including zinc, used and the concentration added in the product label as described in Regulation 2009/767/EU [65].

The most recent regulation of the European Council (Regulation 2016/1095 of 6 July 2016) sets a maximum of 200 mg/kg as is of zinc for supplemented dog foods (≈227 mg/kg DM, assuming 12% of moisture), which represents a decrease of 20% compared to the previously authorized level, 250 mg/kg as is [64]. Although the previously authorized zinc level was considered safe for target species, the release of zinc into the environment was considered an unavoidable risk. Indeed, the accumulation of zinc in soils (mostly in acidic sandy soils) and the leaching from it to surface waters was pointed to as a risk for organisms that reside in soil and water [66].

Table 2 shows the authorized sources for animal feed, which include five inorganic and six organic sources.

### 4.2. Zinc Content in Commercial Dog Foods

**Figure 2:**
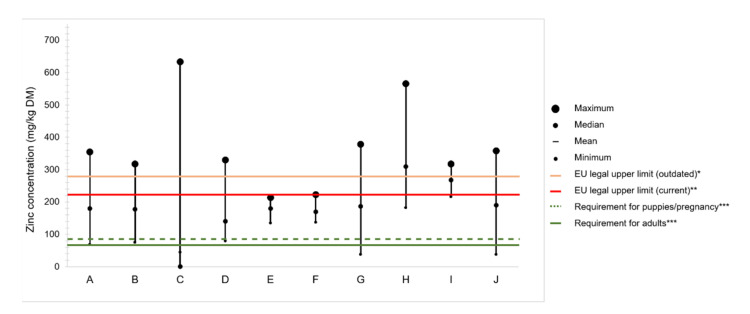
Zinc concentration in commercial dog foods reported in several studies, classified according to the type of food, dry food (DF), wet food (WF); and the age category, puppy (P), adult (A), and senior (S), whenever the information was provided in the publications: A-*n* = 59, DF, P + A [71]; B-*n* = 26, DF, P [72]; C-*n* = 34; DF, P + A + S [73]; D-*n* = 18, DF [74]; E-*n* = 15, DF, P + A + S [75]; F-*n* = 49, WF, P + A + S [75]; G-*n* = 24, DF [76]; H-*n* = 20, DF, A [77]; I-*n* = 6, DF, P [77]; J-*n* = 119, DF, P + A [78]. * Maximum legal limit in the European Union (EU) Regulation No 636/2013; ** Maximum legal limit in the EU Regulation No 2016/1095; *** Zinc requirements published by FEDIAF [66]. DM—dry matter.

A complete compound food is, by definition, a single diet capable of ensuring the animal requirements for energy, macro, and micronutrients [35]. Figure 2 displays a compilation of studies in which the zinc content of complete commercial foods for puppies and adult dogs has been determined. It should be noted the great variability between foods, and the more frequent non-compliance with the maximum authorized limit than with the nutritional requirement.

The eight studies presented in Figure 2 compile zinc determinations of 18 to 119 food samples both for puppies, adult, and senior dogs, mainly commercialized in the United States of America, Europe, and Latin America. Adult formulas were the most share of the samples analyzed, and the results were rarely presented according to age category. Five out of eight, report similar ranges of zinc content, from <100 to ≤378 mg/kg DM. In two studies, the lowest content of 38 mg/kg DM was found [76,78], which corresponds to 50% of the minimum requirement for zinc in dog foods. In addition to dry foods, Davies et al. analyzed 49 wet samples from different brands and found that the content of zinc in wet and dry foods ranged from 137 to 223 and 145 to 214 mg/kg DM, respectively, meaning that both nutritional requirements and legal impositions were respected in all samples [75]. In contrast, Elias et al. reported the greatest variation of all studies, with values ranging from 44 to 633 mg/kg DM [73]. Similarly, zinc excess was reported by Pereira et al., with contents ranging from 248 to 317 mg/kg in puppy and 182 to 566 mg/kg in adult foods [77]. Moreover, the study reported that the zinc content was not affected by the market segment and that the percent of zinc labeled was on median 51.1% of the total content, although in some cases, it was as low as ≈ 20%.

New attitudes and practices of companion animal feeding have emerged in the past years, namely, vegan [79] and raw and homemade diets [80]; thus, zinc content in trendy dog foods has already been reported. Zafalon et al. observed that one of three commercial vegan dog foods analyzed had a content of zinc above the EU legal limits; however, none of the three were below the nutritional requirements, which is somewhat expected since all diets were supplemented with trace element additives [81]. In contrast, the zinc content of home-prepared diets was found below the nutritional requirements in 79% of 75 recipes of dog foods available on the internet [82]. Similarly, Dillitzer et al., who evaluated zinc content in bone and raw food rations, reported that more than half of the samples analyzed failed to supply zinc daily recommended allowance (range 27–400 mg/kg DM; median 76 mg/kg DM) [83]. In their study, the low zinc rations usually consisted of meat with only small amounts of bone and without either offal, zinc-containing supplements, or nuts bone [83].

#### Variation of Zinc Content in Raw Ingredients

The total content of zinc in complete dry dog foods reflects the amount sourced by the raw ingredients (background level), and the amount that is added through supplementation (labeled). Although zinc content of ingredients is available in feed tables, several factors can influence the accuracy of those estimations, being advisable to routinely analyze the content of zinc in raw ingredients. However, that is still not current practice, probably because the analytical techniques require expertise and sophisticated equipment, which inevitably translates into high costs [84].

Plants can be zinc accumulators or not, with tolerance or non-tolerance to high concentrations of zinc in soils [85]. The zinc content of plants depends on the soil characteristics and zinc concentration, and the presence of fertilizers [86]. The zinc uptake by plant roots is facilitated by acidic pH and high content of organic matter of soil, whereas higher phosphorus, iron, aluminum oxides, bicarbonate, clay, and alkaline pH decrease zinc solubility and mobility [87]. In general, plant roots secrete reductants, organic acids (acting as chelators), and H^+^ ions to enhance zinc solubility, and this element is mainly taken up from soil as Zn^2+^ [85]. From the roots, zinc is transported through the epidermis, cortex, endodermis, and pericycle until it reaches the xylem to be mobilized to the shoots [85,88]. The distribution of zinc in plant tissues depends upon the plant species, maturity, and zinc status. If the level of zinc in plants is low to adequate, zinc is mainly found in growing tissues (roots, vegetative shoots, and reproductive tissues), whereas in plants that tolerate zinc at toxic levels, zinc is accumulated in cell walls or vacuoles in the root cortex or leaves [88].

In animal by-products, a great variation in zinc content is often observed as zinc retention varies among tissues. For instance, Henry et al. reported that the content of zinc was 87, 94, 119, 126, and 146 mg/kg DM, respectively, in the heart, spleen, liver, kidney, and muscle of sheep fed diets non supplemented with zinc [89]. Also, the concentration of zinc in bone, heart, and muscle did not respond to an increase of zinc supplementation (700, 1400, and 2100 mg/kg) for 30 days, whereas the concentration in the spleen, kidney, and liver increased, having in kidney and liver been proportional to increment of zinc supplemented [89]. In goats, the zinc content of bone and muscle was 87 and 122 mg/kg DM, respectively [90], and in broilers, the content of zinc in tibia ash, fresh breast muscle, and fresh liver was c.a. 360, 8.7, and 31 mg/kg, respectively [91], when fed the requirement of zinc for each species. Thus, animal species, presence/proportion of offal/bone, and feeding regime of animals comprising the meat meals affect the zinc content of the raw ingredients from animal origin.

### 4.3. Bioavailability of Zinc Sources

The bioaccessibility of a nutrient comprises the fraction released from the food matrix into the intestinal lumen, ready to be absorbed. Bioavailability refers to the bioaccessible fraction that is absorbed through the intestinal mucosa and effectively reaches the bloodstream [92]. In addition to the inherent animal variables (e.g., preexisting tissue reserves, physiological state, disease, and genetics), the bioavailability is affected by the zinc source [33], as it determines how the molecules interact with the conditions it encounters, e.g., the pH and presence of antagonistic compounds in the gastrointestinal compartment [93].

The intestinal absorption of zinc molecules first requires a dissociation that, in the case of salts (inorganic sources), easily occurs in the acidic pH of the stomach. Then, the dissociated cation (Zn^2+^) may bond to amino acids from the chyme or to carrier proteins, embedded in the luminal membranes of the mucosal cells to be transported across the intestinal membrane [26]. However, the dissociated cation may also interact with food components, such as phytates, and eventually form insoluble complexes that lead to the excretion of zinc in feces [13], or with other mineral elements, e.g., calcium, copper, and iron that can interfere with Zn^2+^ absorption and vice-versa [94]. Conversely, if elemental zinc is bond to an organic molecule (zinc chelate), it will not, in theory, dissociate so easily before intestinal absorption, being less prone to precipitate and form insoluble complexes with food components [26]. The absorption of intact zinc chelates might be carried out by zinc transporters, but also by peptide and amino acid transport mechanisms. According to an in vitro study performed with Caco-2 cells, zinc methionine regulates the mRNA expression of ZIP4, ZnT1, and PepT1, suggesting that this chelate is transported either by the traditional zinc ion channel or by the small peptide transport pathway (PepT1) [95]. The permeation of peptides is affected by the chain-length and sequence of the peptides, whereas PepT1 and the proton gradient (the driven force for PepT1 mediated transport) are affected by dietary and pharmacologic compounds (e.g., flavonoids, fatty acids) [96]. In that sense, the absorption of zinc chelates is likely influenced by organic molecules, as it might share their mechanism of uptake.

#### Supplementation Strategies to Enhance Zinc Bioavailability

The study of zinc bioavailability sources draws strategies of zinc supplementation in dog foods. Table 3 summarizes studies that evaluated sources of dietary zinc supplementation in dog foods. Overall, the results show differences between inorganic forms and have pointed benefits of organics over inorganics.

A comparison between three inorganic forms of zinc (zinc oxide, zinc acetate, and zinc sulfate) performed by Ozpinar et al., showed higher apparent absorption of zinc sulfate, although zinc concentration in blood, urine, and feces was not different among sources [5].

Wedekind and Lowry evaluated the bioavailability of zinc oxide and zinc propionate under three conditions: (1) Recommended dietary level of calcium, (2) high calcium level, and (3) high calcium and fiber level, under the assumption that those conditions would, by that order, compromise zinc bioavailability [8]. Multiple regression slope-ratio analysis between zinc intake and plasma concentration of zinc revealed that zinc propionate was more bioavailable. Moreover, calcium reduced the bioavailability of both forms with less impact on zinc propionate. Retention of zinc in declaws, teeth, and testis was unaffected by the source of supplemental dietary zinc. However, the intake of zinc was different due to variation in zinc content of diets that averaged ≈46 mg/kg for zinc oxide and 76 mg/kg for zinc propionate, precluding a direct comparison of zinc bioavailability. Indeed, tissue retention may not be linear, and in that case, the low ratio of tissue concentration:intake of zinc propionate would indicate the worst bioavailability for this source.

Lowe et al. studied the absorption of zinc oxide and zinc amino acid chelate, concluding that the organic form was twice as bioavailable as the inorganic [10]. Zinc amino acid chelate (methionine and glycine) promoted higher hair retention and growth rate when compared to zinc oxide and zinc polysaccharide [9]. Moreover, the negative effect of dietary calcium was only noticed for zinc oxide, increasing its fecal excretion, and decreasing the quality of hair parameters.

According to Lowe and Wiseman, zinc sulfate is more bioavailable than zinc oxide but less available than zinc amino acid chelate (methionylglycinate) [7], which also agrees with data of Jamikorn and Preedapattarapong [41]. Indeed, serum alkaline phosphatase activity, hair growth, zinc deposition, and absorption were higher for dogs fed diets supplemented with zinc amino acid chelate than with zinc sulfate [7]. It must be emphasized that the absorption of zinc reported in this study (37% and 29.8% for zinc amino acid chelate and zinc sulfate, respectively) was calculated from total fecal content without considering the endogenous losses.

Pereira et al. tested the supplementation of zinc sulfate and zinc proteinate at an adequate level in dog foods with high phytate content, with and without the concomitant addition of an enzymatic complex to degrade phytates, which are known to affect zinc bioavailability [13]. Results showed that zinc proteinate was associated with higher bioavailability of phosphorus and a higher percentage of circulating CD4^+^ T-cells suggesting an improved T-cell differentiation. However, no other biomarkers (e.g., plasma and hair zinc concentration, coat quality, serum SOD activity) responded to the zinc source [13]. These results are in line with another study in which weaning puppies were fed a control diet with a background level of zinc of 50 mg/kg and compared to four other dietary treatments consisting of a control diet supplemented with 50 or 100 mg/kg of zinc, either as zinc oxide or zinc methionine [12]. Although weight gain and plasma zinc concentration were lower in dogs fed the control diet (zinc 50 mg/kg) in comparison to the other four dietary treatments, only total alkaline phosphatase was affected by zinc source, being higher in dogs fed zinc methionine [12]. Both studies highlight the need to find more sensitive zinc biomarkers, that can differentiate the bioavailability of zinc sources within adequate zinc status.

Another aspect to be considered when studying mineral supplementation is the source of all supplemental mineral elements. Trevizan et al. compared the total replacement of essential inorganic trace elements (zinc, manganese, copper, and selenium) by an organic source consisting of a 2-hydroxy-4-(methylthio)butanoate (HMTBa) complexed with zinc, manganese, copper, and selenium and observed that despite blood zinc and zinc retention in hair were unaffected, the total replacement of inorganic elements improved immunity by preventing the decrease in antibodies against sheep red blood cells, and improved coat quality [11]. The benefits of replacement of inorganic trace elements by chelated also extend to reproductive performance. Female dogs fed chelated zinc, manganese, and copper during estrus, pregnancy, and lactation (up to the 6th week) had more pups than the ones fed inorganic zinc, manganese, and copper [6]. However, no differences were reported in bodyweight of both damns and puppies nor in the concentration of zinc in hair, although damns fed chelated trace elements had smoother and less fragmented hair follicles, only visible through scanning electron microscopy [6].

The above-mentioned studies seem to point towards a positive effect of organic zinc regardless of the type of chelate (e.g., proteinate, amino acid) in comparison to inorganic, even though some reports are contradictory. Indeed, chelates behave differently in the gastrointestinal tract, as their stability depends upon several factors, e.g., pH, temperature, ionic strength, and characteristics of the ligand [26]. For instance, the stability of the bond between the amino acids and proteins is variable, and if only moderately strong, they might dissociate at pH values lower than 3 or higher than 9, being in those conditions as susceptible as inorganic minerals [93]. Additionally, the ligands are susceptible to different digestive processes, which conditioned the bioavailability of the chelate. For instance, peptide-chelates are susceptible to pepsin action, whereas amino acid chelates do not have peptide bonds to be targeted. This is relevant since some peptides are absorbed and are bioactive as is, but others require dissociation of peptide-zinc complexes to release the metal for absorption. In that case, it is not advantageous when a high molecular weight ligand with high-chelating zinc capacity, resists peptide digestion, and thus, prevents the absorption of zinc [97].

It seems consensual that the effect of zinc source becomes more evident after a restriction period of dietary zinc, likely due to the alteration in zinc pools and status. If so, the differences in zinc status at the beginning of each of the supplementation studies might contribute to the contradictory results observed. Nevertheless, and as previously stressed, more sensitive biomarkers are required to precisely evaluate the zinc sources in dogs at different physiological stages, without the need to deplete zinc status.

The use of zinc nanoparticles (zinc oxide) for supplementation of animal feed has been documented in other species, with improved absorption and quality of animal products [98,99]. However, to the authors’ knowledge, the use of zinc nanoparticles has not yet been reported to improve zinc bioavailability in dog foods, being, therefore, a topic suggested for future research.

A fine supplementation strategy ensures the efficacy of products with benefits for dogs and improves the product at a technical level, optimizing the cost and complying with both nutritional requirements and legal impositions.

## 5. Role of Zinc Status in the Occurrence of Disease

Many disorders have been associated with inadequate zinc status. However, it is often not clear whether the occurrence of disease is the consequence or the cause of poor zinc status.

Zinc deficiency might be explained by insufficient intake (e.g., anorexia, low dietary content in food, poor bioavailability from foods, compromised intestinal absorption) or a defect in metabolism and zinc distribution caused by dysregulation of homeostasis mechanisms (e.g., defects in zinc transporters ZIP and ZnT, in binding proteins MT) [100]. In turn, little is known regarding zinc excess, and the available data in dogs is nearly strict to reports of zinc intoxications, a consequence of inadvertent ingestion of foreign bodies.

The following sections present an overview of diseases in which altered zinc status was reported.

### 5.1. Skin Disorders

The skin of dogs is structured into three layers, the hypodermis (composed mainly of adipose tissue), the dermis (middle layer), and the epidermis (outermost layer). The dermis nourishes and supports the epidermis and other skin appendages (e.g., hair follicles and sweat glands) through anatomical components, such as vessels that arise from the hypodermis, sensory nerves, and immune cells to protect against pathogens [101]. Most dogs’ body surface is covered by hair; therefore, the epidermis is much thinner than in human skin, with only three layers of living cells. In turn, the epidermis of unhaired skin (e.g., planum nasale, at the mucocutaneous junctions, and footpads) is thicker and consists of several layers of living keratinocytes [101]. Melanocytes, Langerhans cells, Merkel cells, and epidermal-resident memory T cells, are also present in the epidermis [101]. Although not available in dogs, studies in other mammals showed that zinc transporters are present in skin cells of the epidermis, having an important role in health and skin homeostasis [102]. For instance, ZIP4 and specially ZIP2 are highly expressed in keratinocytes and are involved in their proliferation [103]. Moreover, ZIP10 is highly expressed in the epidermal progenitor cells located in the outer root sheath of hair follicles and plays an important role in zinc regulation for the maintenance of the skin epidermis [104]. In vitro studies showed that ZIP2 was upregulated by the differentiation induction of cultured keratinocytes and that when this transporter was knocked out, the differentiation was inhibited [105].

#### 5.1.1. Lethal Acrodermatitis

Lethal acrodermatitis, an autosomal recessive hereditary disease typically seen in Bull Terrier dogs, has been associated with acrodermatitis enteropathica, a rare autosomal recessive disease of humans caused by a mutation of a gene that encodes expression of ZIP4, affecting both intestinal absorption of zinc and Langerhans cells [106]. Young dogs carrying the disease may exhibit anorexia, poor growth, impaired immune responses, and skin lesions located in the face, head, and paws, compatible with parakeratotic hyperkeratosis [107], concomitant with low serum zinc [108,109]. However, the association of lethal acrodermatitis with humans’ acrodermatitis enteropathica appears to be misleading, since a defect on ZIP4 has not yet been reported in affected dogs. Contrarily, a defect in the *MKLN1* genes, that encodes muskelin 1, an intracellular protein not yet associated with zinc, was identified as a possible cause for lethal acrodermatitis [110]. In this sense, it remains unclear the connection between lethal acrodermatitis and low zinc status found in dogs, which justifies further research.

#### 5.1.2. Canine Zinc-Responsive Dermatosis

Canine zinc-responsive dermatosis is a well-documented disorder in which, according to Columbini, two distinct syndromes may be present [16]. Syndrome I occurs in dogs of all ages, with a greater predisposition of Northern breeds, although it has also been reported in a study with 16 Boston Terriers [111]. This syndrome has been associated with a genetic defect that impairs intestinal zinc absorption which increases zinc requirements. In turn, Syndrome II has been associated with a low level and/or bioavailability of zinc in diets. It occurs mainly in puppies regardless of the breed and might be due to higher zinc requirements for growth and development. According to White et al., who studied the disorder (both syndromes) in 41 dogs of several breeds, the lesions were ranked by incidence as follows: Crusts, alopecia, pruritus, and erythema in several locations (e.g., periocular region, perioral region, foot-or carpal-pads, pinnae, nasal planum, perigenital region, muzzle, distal limbs, perianal region, and elbow) [14]. Other lesions reported in five Labrador Retrievers with zinc-responsive dermatitis included ceruminous otitis externa and superficial lymphomegaly accompanied by dry hair coat, mild generalized seborrhea sicca, with concomitant low levels of serum and hair zinc [112]. In most cases, skin biopsies revealed parakeratotic or multifocal orthokeratotic hyperkeratosis, epidermal and follicular acanthosis, and inflammatory infiltrates with different types of cells [14,111]. Available studies did not evaluate the expression of zinc transporters and MT in skin cells that would be useful to better understand both syndromes and the origin of the keratinization defects.

Although non-zinc responsive dermatosis can present lesions similar to zinc-responsive dermatosis, zinc concentration in serum and hair is only significantly lower in the latter, highlighting the importance of this parameter for the diagnosis [113]. Along with low serum zinc, dogs with zinc-responsive dermatosis present increased levels of serum malondialdehyde and decreased activity of SOD and catalase in comparison to healthy individuals [114], both markers of oxidative stress and antioxidant activity. Zinc has an important antioxidant effect that it can carry (1) in its ion form (e.g., by competing with other potentially toxic metals, and thus, contributing to cell membrane stabilization), (2) through its structural role in proteins, (3) by modulating MT induction, (4) through protecting protein sulfhydryl groups from oxidation, and (5) by acting as a cofactor of scavenger enzymes, such as SOD [61,115]. Indeed, a study by Romanucci et al. showed that MT immunoreactivity in the epidermis of dogs with zinc-responsive dermatosis was absent, indicating low local levels of zinc, and thus, higher susceptibility to oxidative stress [116]. Moreover, high levels of heat shock proteins were found, which appears to be a mechanism for protection against oxidative stress, inhibiting apoptosis, as well as cell cycle regulation of proliferating keratinocytes, resulting in acanthosis and parakeratosis in zinc deficiency [116].

Zinc is a cofactor of the enzyme delta-6 desaturase, that catalyzes a reaction step in the biosynthesis of linoleic acid into arachidonic acid [117], which is then taken by keratinocytes and used for the formation of a functional epidermis [118]. According to van den Broek and Simpson, dogs with zinc-responsive dermatosis had low quantitative absorption of long-chain triglycerides, before and up to four hours after feeding vegetable oil [119]. This might explain why poor zinc status can impact essential fatty acids metabolism, and therefore, aggravates skin lesions. Furthermore, Marsh et al. reported a synergic effect of linoleic acid and zinc supplementation in healthy dogs, with an improvement in coat gloss and coat scale, and a decrease in the transepidermal water loss, which highlights the role of zinc in promoting the health of skin and coat [15].

#### 5.1.3. Canine Atopic Dermatitis

Canine atopic dermatitis is a chronic genetically predisposed, inflammatory, and pruritic skin disorder, that likely accompanies dogs throughout life [120]. McFadden et al. reported that dogs affected with the disease improved clinical signs (lower Canine Atopic Dermatitis Lesion Index) by receiving allergen treatment (glucocorticoids or ciclosporin) associated with a zinc-containing supplement (zinc methionine, eicosapentaenoic acid, docosahexaenoic acid, and biotin) when compared to dogs receiving the same medication associated to an identical supplement but without zinc methionine [121]. Moreover, dogs that received glucocorticoids combined with the zinc-containing supplement, presented less pruritus, and a reduction of medication was possible for at least four weeks, comparing with dogs receiving ciclosporin and the zinc-containing supplement, which suggests that glucocorticoids enhance zinc absorption [121]. The benefit reported could be attributed to a synergy between zinc and essential fatty acids, but also biotin. Biotin is a water-soluble vitamin that showed a positive impact on dogs with skin diseases [122], and which deficiency has been related to low zinc levels in humans [103]. Nevertheless, the benefit of zinc in canine atopic dermatitis could also be due to its role in the mechanisms of skin inflammation. A study by Hayashiya et al. (2002) reported that peripheral blood mononuclear cells of dogs with atopic dermatitis tended to express a type 2 cytokine pattern (interleukin, IL-4, IL-5, and IL-10) and decreased type 1 profile (interferon-gamma) [123]. Indeed, an imbalance of T-helper (Th1):Th2 cell ratio is associated with zinc deficiency, as its flux and homeostatic zinc signals are crucial for proper T-cell differentiation [124]. This suggests that zinc deficiency contributes to the disruption of skin homeostasis as the Th2-type immune response downregulates the expression of proteins involved in the formation and maintenance of the epidermal barrier (e.g., filaggrin, involucrin) [106]. Olivry et al. reported dendritic cell hyperplasia and higher percentages of IgE+ cells in canine atopic dermatitis and increased epidermal Langerhans cell counts in lesional specimens suggesting an epidermal allergen contact that indicates a role of antigen-presenting cells in the pathogenesis of the disease [125]. Even though serum IgE of dogs with canine atopic dermatitis was not different from healthy dogs [126], in humans, decreased serum zinc levels are associated with increased total IgE levels and allergic sensitization [127], which might justify further study of the correlation of serum zinc and IgE levels in dogs with allergy. Moreover, Langerhans cells were implicated in the pathogenesis of canine atopic dermatitis and in the expression of class II major histocompatibility complex in dendritic cells [103]. As zinc is crucial for Langerhans cells, it may suggest that zinc status might play a role in canine atopic dermatitis, although there are no studies addressing its involvement. Yet, the search for therapeutic approaches for canine atopic dermatitis has increased over the years, e.g., naturally occurring polyphenolic flavones, such as luteolin, that target keratinocyte cytokines appears promising [120]. This highlights the need to further study the benefits of associating zinc supplementation with nutraceuticals, as it induces fewer side effects for long-term treatments.

#### 5.1.4. Symmetrical Lupoid Onychomadesis

Symmetrical lupoid onychomadesis is a disorder of Bearded Collies, as well as other breeds, in which dogs present onychomadesis with detachment and subsequent shedding of one or more claw plates, that regrow short, dry, deformed, and brittle [128]. Affected dogs present low content of zinc in claws in comparison to healthy individuals, though concentration in hair is not altered. Supplementation of essential fatty acids constitutes a therapeutic approach in combination with antibiotics [129]. Due to zinc’s role in the keratinization process and metabolism of essential fatty acids, zinc status might be associated with the development of the disease, although no data is available.

### 5.2. Neurological and Behavioral Disorders

It was reported that serum zinc levels were higher in epileptic dogs [130]. Although serum zinc does not necessarily correlate with total zinc in the brain, due to the existence of control mechanisms independent of peripheral zinc level [131], this suggests a role of zinc in the pathophysiology of epilepsy in dogs. Adequate levels of zinc in the central nervous system confer neuroprotective activity, whereas high concentrations can be neurotoxic, being zinc homeostasis crucial to brain and systemic physiology [131]. In the central nervous system, zinc might be bound to zinc-dependent enzymes and other proteins (e.g., transcription factors and MT), or in its free form (Zn^2+^), mostly in presynaptic vesicles of glutamatergic neurons [132]. In homeostasis, zinc is released from the synaptic vesicles and modulates both ionotropic and metabotropic postsynaptic receptors, whereas, under clinical conditions (e.g., epilepsy), the excessive influx of zinc into neurons can damage the postsynaptic neurons [131]. Moreover, MT distributed in the central nervous system are responsible for zinc homeostasis and also constitute an antioxidant defense mechanism, reducing neuroinflammation and oxidative stress, which have been pointed to as major causative factors of neurodegenerative diseases [133]. Indeed, inducers of oxidative stress activate autophagy, and zinc dysregulation appears to be involved in the process [115]. Under physiological conditions, autophagy is responsible for the degradation of abnormal protein aggregates and waste organelles and thus, important for cell survival [134]. However, downregulation of MT-3, predominantly expressed in the brain, during injurious oxidative stress reduces the release of zinc, preventing excess zinc accumulation, reducing the autophagy flux and cell death [135]. Although in humans and animal models, zinc status has been associated with impaired brain development, neurodegenerative disorders, as well as neuronal damage associated with traumatic brain injury, stroke, and epilepsy [132], studies in dogs are still scarce.

Impaired social behavior has already been associated with zinc deficiency in rodents and monkeys [136], and in dogs with behavior problems (e.g., excessive activity, aggression towards people and dogs, destructiveness, inappropriate elimination, and fearfulness). These dogs present significantly lower serum zinc levels in comparison to dogs without behavior problems [137]. A study by Rahimi et al. indicated that supplementation with zinc, magnesium, and gelatin capsules of fish oil supplement (containing eicosapentaenoic and docosahexaenoic acid) reduced behavior problems of dogs, namely, fearfulness, destructiveness, and inappropriate elimination [138]. In this study, the therapeutic effect of zinc was not isolated, therefore it would be important to do so to comprehend the link between zinc status, behavior, memory, and learning.

### 5.3. Eye Disorders

Zinc is considered to be essential for optimal retinal cell metabolism, modifying photoreceptor plasma membranes, regulating the light-rhodopsin reaction, and modulating synaptic transmission [139]. In mice, ZnT3 and ZnT7 transporters, present in different layers of the retina, along with MT, ensure zinc homeostasis and participate in ocular functions [139]. Moreover, zinc is essential to maintain levels of taurine in the retina acting on TAUT, a transporter responsible for the translocation of taurine in tissues [140]. In cats, lower levels of taurine produced marked abnormalities of rod and cone b-wave amplitude and timing exhibited in electroretinograms, as well as a fundal lesion (mainly in area centralis that progressed from hyperpigmentation to an elongated hyper-reflective scar) [141]. In healthy dogs, taurine levels in aqueous humor average ~50 mM [142], and a decrease in taurine concentration causes damage in the photoreceptors and cell death in dogs with primary glaucoma [143].

Eyeshine is a phenomenon of light reflection from the tapetum lucidum, characteristic of some species, including dogs. Zinc is a component of the tapetum in healthy dogs, however, in abnormally shaped tapetal cells from Beagles with an inherited autosomal recessive trait, zinc was found lower to absent [144]. The decrease of tapetal zinc content has also been attributed to zinc chelators, such as ethambutol, which results in ultrastructural disorganization and loss of tapetum color [145].

### 5.4. Reproductive Disorders

The importance of zinc in reproductive performance is well documented in males [146]. In dogs, seminal plasma contains zinc ions that play structural and regulatory roles in the activity of prostate-specific arginine esterase, which maintains a normal function of the prostate and spermatozoa [147]. A work by Alonge et al. showed that a commercial dry diet, including a complex of vitamin E (250 mg/kg), selenium (0.27 mg/kg), zinc (180 mg/kg), folic acid (1.5 mg/kg), and omega-3 polyunsaturated fatty acids (5 g/kg) increased the number of spermatozoa, and improved motility and membrane properties of the ejaculate in healthy normospermic dogs [148]. Moreover, dogs suffering from infertility were given a nutraceutical diet with balanced omega-6:omega-3 ratio (4:1), enriched with zinc (50 mg/kg), *Lepidium meyenii* (865 mg/kg), *Tribulus terrestris* (52 mg/kg), l-carnitine (420 mg/kg), beta-carotene (230 mg/kg), vitamin E (240 mg/kg), and folic acid (0.27 mg/kg), that increased motility percentage, semen volume and concentration, and the total number of sperms per ejaculation [149]. However, in both studies, the effects of zinc were not isolated. Nevertheless, the addition of prostasomes (extracellular vesicles released into the prostatic fluid by prostate epithelial cells with a high content of zinc ions) to ejaculates collected from dogs, had a positive effect on the sperm plasma membrane and acrosome integrity, which highlights the role of zinc in sperm function and in enhancing the quality of it during cold storage for insemination [150].

Conversely to males, the effect of zinc in the reproductive performance of females is scarcely reported. White et al. suggest that during estrus, the ovarian tissues demand high levels of zinc, which lowers the status of zinc and its availability for other tissues, thus increasing the possibility of the appearance of pathologies, such as zinc-responsive dermatosis [14]. This hypothesis has yet not been confirmed, nor the exact role of zinc in the reproductive tract of females was defined. Nevertheless, it is known from studies in mice that zinc is essential during pregnancy for adequate placental morphogenesis and maternal blood pressure adaptations [151]. In pregnant dog females, a positive association between litter size and dietary zinc bioavailability was reported [6], however further studies must enlighten the role of zinc in the reproductive tract of females.

### 5.5. Disorders Related to Natural Infections Caused by Pathogens

Panda et al. (2009) reported that puppies with hemorrhagic gastroenteritis, caused by canine parvovirus, had lower blood zinc and higher erythrocyte lipid peroxide levels and catalase activity compared to healthy individuals [152]. Similarly, dogs with babesiosis, caused by natural infection of *Babesia gibsoni*, exhibit higher erythrocytic lipid peroxide levels and activities of SOD and catalase concomitant with low zinc blood levels [153]. Another example is canine hepatozoonosis, a disease caused by a parasite, *Hepatozoon canis*, that infects the host mainly through a tick, *Rhipicephalus sanguineus*. Besides several hematological and biochemical abnormalities, hepatozoonosis is also associated with low serum zinc [154]. The low zinc status in these examples might be related to a decrease in food consumption, since anorexia is a frequent clinical sign of many infections. However, in parvovirus, the low zinc status might also be due to diarrhea, as it prevents the reabsorption of zinc that under physiological states takes place after pancreatic and biliary excretions [155]. In that sense, it is likely that regardless of the etiology, enteritis associated with persistent diarrhea could affect zinc status. Indeed, that hypothesis is in line with a report that described low serum zinc in dogs affected with inflammatory bowel disease [156].

Another point to consider is that, during an infection, serum zinc levels are often markedly reduced, as the host organism sequesters zinc and other nutrients to restrict its availability to pathogens [157]. This is accomplished through the expression of pro-inflammatory cytokine IL-6 that upregulates the expression of zinc-binding transporter ZIP14 and peptides, such as MT and A2M [124,157]. *Leishmania*, a mandatory intracellular parasite, requires zinc for survival and proliferation, thus zinc depletion by a disruption in its homeostasis resulted in apoptosis-like cell death of the parasite [158]. Symptomatic leishmanial-infected dogs presented marked alteration in serum biochemistry (e.g., lower serum total antioxidant status and albumin concentration, with a concomitant increase in serum malondialdehyde, blood urea, and nitrogen concentrations), when compared with both healthy and asymptomatic leishmanial-infected dogs. Both asymptomatic and symptomatic leishmanial-infected had low serum zinc, particularly in the symptomatic group [159]. The decrease in zinc status observed in both infections could be due to nutritional immunity mechanisms for limiting zinc bioavailability, rather than just a consequence of insufficient intake related to the progression of the disease, as above suggested for general infections.

### 5.6. Hepatic and Renal Disorders

Zinc is predominantly stored in the liver [45,160,161], whereas the biliary system constitutes a major route of zinc excretion and enterohepatic circulation [100].

It was observed that dogs with pathological liver lesions had a significantly lower zinc liver content, comparing with dogs without macroscopic abnormalities [160]. That might be related to MT, which bonds zinc to protect cells, being upregulated by zinc excess but also by other mechanisms [100]. Indeed, MT are also upregulated by food and water deprivation, which may occur due to anorexia associated with liver disease, by cytokines and inflammatory mediators (e.g., IL-1, TNF, IL-6), general inflammation, stress, and drug administration, such as glucocorticoids [100]. The upregulation of MT in the absence of zinc excess will cause zinc status to decrease, aggravating zinc deficiency. Additionally, a relatively high concentration of zinc-MT in the liver seems to be required for hepatoprotection, being released in response to inflammation and redox imbalance [100].

Copper-associated hepatitis is a hereditary disease described in some breeds (e.g., Labrador Retrievers and Bedlington Terriers) in which copper accumulation precedes the appearance of clinical signs of inflammation [161]. Affected dogs present respectively low and high liver concentrations of zinc and copper [162], and high levels of liver MT bound to copper instead of zinc [163]. In squirrels, it was proposed that MT binds copper to protect against its excess, but when the amount of accumulated copper exceeds the capacity of cells to synthesize MT, zinc-bound MT can act as an antioxidant via replacement of the zinc with copper [164]. Copper-associated hepatitis therapy intends to reduce copper absorption in the gastrointestinal tract by given low-copper diets or enhancing copper excretion with the administration of copper chelators, such as d-penicillamine or trientine [45]. As zinc induces expression of MT in enterocytes, which binds to copper, dietary zinc supplementation has also been used to decrease copper status [165,166]. The copper-metallothionein complex remains in the enterocyte, preventing it from entering the blood circulation, being thereafter excreted back to the luminal gut [45].

Little is known about the impact of renal disorders on the zinc status of dogs. A study by Passlack et al. reported that the occurrence of chronic kidney disease (CKD) did not affect the storage of zinc in the kidneys nor the liver of dogs [46]. Zinc net reabsorption is the dominant tubular mechanism for renal handling that prevents its excretion [44]. In that sense, CKD can likely enhance zinc losses in urine, and thus, lower the status [167]. Furthermore, a study in humans found a relation between CKD incidence rate and low dietary zinc [168], and another highlighted the benefits of its supplementation for patients undergoing maintenance hemodialysis [169]. These associations may suggest a link between CKD and zinc status, which should be further evaluated.

### 5.7. Cancer

The role of zinc status in canine cancer is scarcely reported. However, Brodziki observed significantly higher zinc concentration in both malignant and benign skin neoplastic tissues of dogs, compared to controls [170]. This was attributed to two possible causes (1) the intense metabolic processes in neoplastic cells and enhanced activity of intracellular enzymes that require intracellular zinc for proper function, or (2) the increase of intracellular zinc that inhibits neoplastic cell apoptosis [170]. Conversely, zinc was substantially lower, while copper substantially higher, in the neoplastic tissue of dogs with hepatocellular carcinoma [171] and females with mammary tumors [172], compared to healthy tissues. This might be explained by zinc chelation, which is particularly important in this context since copper is essential for angiogenesis, and by lowering copper tissue levels, zinc might limit tumor growth [173].

Serum zinc was reported to be lower in dogs with lymphoma and osteosarcoma [174]. Moreover, dogs with malignant perianal tumors had low serum zinc and high total antioxidant capacity before treatment and, after six months of treatment, serum zinc increased, and total antioxidant capacity decreased [175]. Low serum zinc levels might be related to a catabolic state and increased frequency of cachexia, a condition that often accompanies cancer [174], or to zinc distribution into neoplastic tissues. This latter hypothesis is in line with the work of Dash et al., who observed decreased levels of zinc in plasma of females with mammary tumors, yet higher values in the neoplastic tissue compared to healthy individuals [176]. In this study, the significantly lower values in plasma zinc concentration were only present when dogs were fed a homemade diet, probably poor in zinc as dogs fed a balanced commercial diet had significantly higher zinc in neoplastic mammary gland tissue, with plasma concentration similar to healthy dogs [176]. This observation highlights the role of proper mineral nutrition in maintaining pool reserves that could prevent zinc deficiency in altered physiological states.

### 5.8. Zinc Poisoning

There is no official maximum tolerable level of zinc for dogs, though for rodents and poultry, the value is 500 mg/kg, and for swine 1000 mg/kg feed [177]. Available studies on zinc toxicity are mainly reports of zinc poisoning, due to the ingestion of foreign bodies (e.g., coins, toys), and, in those cases, the dose ingested is hardly impossible to determine.

The ingestion of foreign bodies is common in dogs of various ages and breeds, though puppies are more prone to exhibit this behavior, which might constitute an additional risk, as their inferior bodyweight imposes lower tolerance levels. According to Gurnee and Drobatz, who reviewed 19 cases of zinc intoxication, the most common clinical signs, by order of frequency, are anemia, vomiting, pigmenturia, hyperbilirubinemia, lethargy, decreased appetite (anorexia), and diarrhea [178]. Hemolytic anemia resembling an immune-mediated was often present, thus laboratory testing might reveal regenerative anemia, hemoglobinemia, hemoglobinuria, and, in some cases, Heinz bodies and spherocytes [179,180]. Lethargy and gastrointestinal signs (vomit and diarrhea) are likely to reflect abdominal discomfort, due to irritation and possibly ulceration caused by the physical presence of the foreign body or the release of zinc, facilitated by the acidic environment of the stomach [178]. Bilirubinemia is associated with hemolysis, but also with liver dysfunction, which can also be reflected by high serum activities of liver-associated enzymes (e.g., alkaline phosphatase, alanine aminotransferase) [180]. Indeed, the extension of liver lesions, the occurrence of pancreatitis (pancreatic fibrosis and acinar necrosis), coagulopathies [178], and acute kidney failure (associated with diffuse tubular degeneration with focal epithelial necrosis) [181] dictate the prognosis of zinc toxicosis. In addition to the clinical symptoms described, the diagnosis is usually achieved through radiographic visualization of the foreign body and determination of zinc in serum [180,181,182]. The treatment of zinc toxicosis aims to remove the foreign body (e.g., surgery or emesis if adequate), provide stabilization and support (e.g., intravenous fluids administration for rehydration, whole blood or specific blood constituents transfusion depending on the severity of anemia and coagulopathy), administration of antibiotics, gastroprotection drugs, and zinc chelators [178]. According to van der Merwe and Tawde, the administration of antiacids on the onset of zinc toxicosis treatment is beneficial as it lowers the gastric acid secretion, increasing pH, and therefore, the rate of zinc dissolution and intestinal absorption [183]. EDTA is a chelating agent that reduces the toxic effects of metal excess, therefore its administration constitutes a therapeutic approach for zinc toxicosis, however, it also affects the metabolism of other essential elements [184]. Therefore, the administration of d-penicillamine is a suitable alternative to EDTA for zinc chelation, enabling faster recovery of patients and preventing complications of zinc toxicosis, which in some cases occur after the removal of the foreign body [185].

Reports of chronic ingestion of high doses of zinc in dogs are scarce, however, a publication by Siow described ingestion of up to 125 g of an antiseptic healing cream containing 15.25% w/w zinc oxide over seven days, which was estimated to constitute a daily intake of 386.4 mg/kg bodyweight of elemental zinc [186]. The clinical symptoms resembled the ones described for acute zinc toxicosis, without apparent pancreatitis nor nephrotoxicity associated [186].

## 6. Conclusions

Zinc requirements currently available for dogs were defined using data of few studies and apply to healthy dogs, at puppy and adult life stages. Pet food manufacturers use those requirements as guides to formulate balanced dog foods. However, studies that evaluated the zinc content of commercial dog foods revealed a wide variation, with a small percent failing to provide the requirements and a significant number having content above the EU legal limit, defined for supplemented animal feeds. Therefore, strategies for zinc supplementation of dog foods still have scope for improvement. It appears from studies on dog food supplementation that organic sources are more bioavailable and often associated with benefits for dogs’ health, although the effects reported are sometimes contradictory, and more sensitive biomarkers are required. In that sense, the search must continue toward improved bioavailability with sources that can meet dog requirements with a lower amount of supplemental zinc, thus reducing the negative environmental impact of zinc excess.

As zinc is involved in multiple body functions, alterations of zinc status have been associated with several diseases in dogs. However, the underlying cause of poor zinc status remains often unreported. It is not clear whether altered zinc status is a consequence of (1) insufficient dietary zinc intake, which by affecting zinc status, increased the susceptibility of disease, or (2) the onset of disease, increased zinc requirements, forcing redistribution, and therefore, secondary alteration of zinc status. Besides, defects in zinc absorption, metabolism, or/and distribution, due to acquired or hereditary anomalies, might also negatively affect zinc status. However, these defects are not properly studied in dogs, thus it is often required to extrapolate data from humans and animal models, e.g., mice and rats, to infer the causes. Moreover, apart from clinical reports of zinc intoxications, maximum tolerable levels, and data on chronic high dietary zinc in dogs are not available, which precludes a conclusion on the risk for dogs feed commercial diets with zinc content above the legal limits.

The review highlights the need for further research to fully understand zinc requirements according to physiological states, the effects of zinc sources on dogs’ health, and the underlying causes of poor zinc status found in some diseases.

## Figures and Tables

**Table 1:** Adequate and minimum recommended allowances of zinc for healthy dogs, defined by the National Research Council (NRC) and the European Pet Food Federation (FEDIAF).

	Adult	Puppies	Gestation and Peak Lactation
Recommended adequate Zn allowances ^1^			
mg Zn/1000 kcal ME	15	18.5	24
mg Zn/kg DM	60	75	96
Recommended minimum Zn allowances ^2^			
mg Zn/1000 kcal ME ^3^	18–20.8	25	25
mg Zn/kg DM ^3^	72–83.4	100	100

^1^ According to NRC (2006) [33]. ^2^ According to FEDIAF (2020) [35]. ^3^ Assuming a ME requirement of 95 or 110 kcal per kg of bodyweight. DM—dry matter; ME—metabolizable energy.

**Table 2:** Zinc sources authorized as additives for animal feed supplementation in the European Union.

Source	Authorized Zinc Source	Regulation	Reference
Inorganic	Zinc acetate dihydrate	2016/1095/EU	[67]
	Zinc chloride anhydrous		
	Zinc oxide		
	Zinc sulfate monohydrate/heptahydrate		
	Zinc chloride hydroxide monohydrate		
Organic	Zinc chelate of amino acids hydrate		
	Zinc chelate of glycine hydrate		
	Zinc chelate of protein hydrolysates		
	Zinc bislysinate	2016/973/EU	[68]
	Zinc chelate of hydroxy analog of methionine	2010/335/EU	[69]
	Zinc chelate of methionine sulfate	2019/1125/EU	[70]

**Table 3:** Studies of zinc source supplementation in dog foods.

Design/Duration/Zn Restriction ^1^	Subjects	Zn Forms	Level Zn ^3^	Biomarker of Zinc Status	Reference
2 wks length for 3 Zn sources; 3 wks washout between each Zn source/2 wks ^2^	6 male Beagles/ 12 wks	Zinc sulfate	0	Fecal, plasma, and urinary [Zn], **apparent fecal absorption**	[5]
		Zinc acetate	2 mg/kg BW		
		Zinc oxide	4 mg/kg BW		
Randomized block design/35 days/ 5.4 mg/kg feed	42 puppies/11 wks	Zinc oxide Zinc propionate	40 ^4^	Weight gain, **plasma [Zn]**, declaws, teeth, and testes [Zn]	[8]
1 meal test for each Zn form with 1 wk between them/2 wks/ 56 mg/kg feed	4 adult Beagles	Zinc oxide Zinc amino acid chelate	50	Fecal, plasma and urinary [Zn], **AUC**	[10]
6 × 4 randomized block design/25 days per treatment/no restriction	4 adult Beagles	Zinc oxide ^5^ Zinc amino acid Chelate ^5^ Zinc polysaccharide ^5,6^	50	**Fecal [Zn], hair growth rate, hair [Zn]**	[9]
Randomized block design/20 days/30 days/ 56 mg/kg feed	27 adult Beagles	Zinc oxide	50	**Hair growth, hair [Zn]**, serum AP, **AUC_5_**	[7]
		Zinc amino acid chelate	75		
		Zinc polysaccharide	100		
Cross-over design/3 wks 2 wks/58.5 mg/kg DM	4 female Beagles	Zinc sulfate Zinc methionylglycinate	≈61.5	**Hair growth, hair [Zn],** plasma and **fecal [Zn]**, **serum ALT, zinc absorption**	[41]
3 Latin Squares 4 × 4/4 diets, 4 periods of 5 wks/no restriction	12 Beagles/ 1 year	Zinc sulfate ^7^	75	Plasma, hair, and urinary [Zn], serum, **ALT**, SOD activity and CRP, **CD4/CD8 ratio in peripheral blood**, coat quality (brightness, softness, greasiness, and scale) and growth (trichogram), CTTAD, flatulence	[13]
		Zinc proteinate ^7^			
Randomized block design/28 d/50 mg/kg DM (7 days)	30 Hound-cross Mongrel/ 8 wks	Zinc oxide	50 ^8^	Weight gain, hair weight and length, plasma and hair [Zn], liver, cortisol, bone, and **total AP**, concentrations, blood MT gene expression	[12]
		Zinc methionine	100 ^8^		
Randomized block design/30 days/no restriction	18 dogs many breeds/ 2—6 years	Zinc oxide Zinc HMTBa ^9^	40	Coat quality (brightness, looseness, **texture**, greasiness), whole-blood and hair [Zn], antibody against sheep red blood cells	[11]
Randomized block design/reproduction (≈12 wks) ^10^/ no restriction	34 female Beagles/> 1 year + newborn puppies	Zinc oxide	53	BW (dams) and weight gain (lactation from birth to 6 wks), **litter size**, hair [Zn], **hair and root morphology by scanning electron microscopy**	[6]
		Zinc proteinate ^11^			

^1^ I of time and zinc supplied during restriction phase. ^2^ Background zinc (ingredients) not provided. ^3^ Zinc supplemented in food (not the total content); units are mg/kg DM unless otherwise stated. ^4^ Level was tested with three conditions: (1) At 10 g calcium/kg diet; (2) 15 g calcium/kg diet; and (3) 15 g calcium/kg diet + 50 g/kg beet pulp. ^5^ Treatments were duplicated with the inclusion of 20 g/kg of calcium. ^6^ Zinc polysaccharide complex consisting of zinc sulfate complexed with alkali-modified brewers wort. ^7^ Each zinc source was provided in a diet alone and another containing 200 mg/kg of a solid-state fermentation product of Aspergillus niger. ^8^ Zinc added to a control diet with 50 mg/kg DM of the zinc background level. ^9^ Total replacement of the following essential trace elements: Copper, manganese, selenium, and zinc. ^10^ 2 wks before estrus + 1 to 3 wks of gestation (maintenance food intake); 4 to 6 wks of gestation (2× maintenance food intake); 6 wks to parturition (maintenance food intake); lactation (food ad libitum). ^11^ Total replacement of the following essential trace elements: Copper, manganese, and Zinc. wks—weeks; BW—bodyweight; AP—alkaline phosphatase. AUC—area under the curve (plasma zinc profiles after a meal). ALT—alanine aminotransferase. CRP—C-reactive protein. SOD—superoxide dismutase. CTTAD—coefficient of total tract apparent digestibility; MT—metallothionein. The biomarkers that responded to zinc sources are bolded.

## Data Availability

Not applicable to the present work.
